# Pediatric neuro-oncology in Latin America and the Caribbean: a gap to be filled

**DOI:** 10.3389/fonc.2024.1354826

**Published:** 2024-03-20

**Authors:** Rosdali Díaz-Coronado, Rosangela Correa Villar, Andrea M. Cappellano

**Affiliations:** ^1^Pediatric Oncology Department, Instituto Nacional de Enfermedades Neoplasicas, Lima, Peru; ^2^Post Graduated Medicine School, Universidad Peruana Cayetano Heredia, Lima, Peru; ^3^Radiation Oncology Department, Centro Infantile Boldrini, Sao Paolo, Brazil; ^4^Medicine School, University of São Paulo, São Paulo, Brazil; ^5^Pediatric Oncology Department, Pediatric Oncology Institute-GRAACC (IOP-GRAACC)/Federal University of São Paulo, São Paulo, Brazil

**Keywords:** pediatric, brain tumors, LMIC, gap, LATAM

## 1 Childhood cancer and disease burden

From the estimated 400,000children and adolescents who develop cancer each year, 80% live in low-middle income countries (LMIC) and are, unfortunately, responsible for 90% of the deaths in this age group (1, 2). The global outcome disparities are influenced by several factors mostly related to the availability of resources, with underprivileged patients placed “on the wrong side of a pediatric oncology ‘death canyon’”, with less than 5% of global resources for cancer dedicated to this group of patients (3–9).

Caring for children with central nervous system (CNS) tumors is a significant challenge due to the number of resources required, ranging from infrastructure to specialized staff. Frequently, the availability of resources is the difference between success and failure and should move pediatric oncologists worldwide to action (10).

## 2 CNS tumors overview in Latin America (LATAM) and the Caribbean

Latin America and the Caribbean is a diverse region, with countries varying in culture, language, and health systems strength. In 2020, based on GLOBOCAN data, there were 2,585 incident cases of pediatric CNS tumors in Latin America and the Caribbean. Brazil (791), Mexico (702) and Colombia (179) had the most cases. In terms of age-standardized incidence rates, there is a considerable variability in the region, with Guatemala (0.47), Bolivia (0.73), and Panama (0.89) having the lowest rates, while Mexico (2.2), Uruguay (2.3) and Guadeloupe (3.2) having the highest (11).

Data on survival, is a challenge mainly due to the lack of pediatric cancer registries. Despite this, Girardi et al, reported a survival analysis from 2000 to 2014 for LGG, that turns to be uniform between High income countries (HIC) ranging from 90 to 100% compared to a LATAM countries reaching almost 70%.; for medulloblastomas there are variable survival rates ranging from 30 in LMIC, compared to over 80% in HIC. Although we know that there is an under representation for countries classified as LMIC, the real gap is still difficult to calculate so far, but we can imply is wide larger. A preliminary results of a multicenter study on medulloblastoma survival on 4 centers belonging to LMIC (Peru, Pakistan, Philippines and Uruguay) reports a 75% 5-year overall survival and 35% Event-free survival, this example depicts the existent gap in the second most common tumor globally (12, 13).

In pediatric brain tumors, despite the epidemiological distribution of LMIC being seemingly similar to HIC, the well-known vulnerabilities and dismal outcomes are particularly true (14). As the second most common malignancy in childhood and the leading cause of death related to cancer is still a therapeutic challenge (15–18). The cause of high mortality in LMICs is multifactorial, with factors like delayed diagnosis, misdiagnosis, late presentation, late referrals, delay in starting proper treatment, high rates of treatment abandonment that can exceed 20% in LATAM, and comorbidities are contributing elements like high rates of malnutrition that can have adverse effects of treatment tolerability and lower survival. Other causes include limited access to supportive care, including palliative care, essential medications, continuous medical education, and multidisciplinary approach (7, 9, 19–21).

Lack of pediatric cancer registries in LMICs limits the estimation of the real burden of childhood cancers and how each of these factors contribute to the trends in survival and mortality in each country, thus preventing identification of effective interventions. The implementation of a quality registry, an adequate hospital infrastructure with a surgical center equipped for neurosurgery, intensive care unit prepared for highly complex cases and an appropriate multidisciplinary team requires financial resources, time, and expertise (22). In addition, recent advances in the molecular characterization of cancer have promoted considerable changes in diagnosis, prognosis, and outcome for our patients. Thus, the resources and expertise that an institution in a LMIC would need to allocate to characterize such tumors are indeed limiting and not always justified since targeted therapies are also not readily available (23, 24).

## 3 Regional and international interventions

Over the past years, pediatric cancer has gained momentum in the context of cancer control and non-communicable diseases. The World Health Organization (WHO) member states passed a resolution on May 31, 2017, to add cancer prevention and control initiatives for all age ranges (25). In 2018, the WHO launched the Global Initiative for Childhood Cancer (GICC) aiming to achieve at least a 60% survival for pediatric cancer patients by 2030. Importantly, a CNS tumor, low-grade glioma, is included one of the six index cancers selected by the GICC to demonstrate impact of the interventions. The goal achievements will occur through governmental support for awareness of childhood cancer at national and global levels and promoting the capacity expansion of countries to perform best practices in care for these patients (26). Furthermore, the GICC has gained a lot of traction in Latin America with many activities ongoing to improve care capacity (27). In Peru, thanks to the GICC, a multicenter team was created to map the baseline on LGG, the results showed a survival of 80%, and recognized the importance of long-term effects of treatment, information that is not available due to the loss of follow up, and the lack of long term follow up programs (28).

To provide comprehensive cancer care for children with CNS tumors, a strong health system needs to exist, with robust referral networks and specialized centers. In Brazil, a unified national health system (SUS) was implemented in 1990 subsidizing most cancer treatments and the creation of high-complexity cancer centers (CACON). In Mexico, social health insurance was established in 2006, and combined with standard treatment protocols and accreditation of childhood cancer, the treatment abandonment was reduced from 35% to 5% (15, 16). It is anticipated that the GICC will continue to help strengthen pediatric cancer in national cancer control plans in Latin America.

Prompt diagnosis, referral, multidisciplinary approach, and local capacity are important tasks where interventions can improve the outcomes. An example of success due to the effort of well-structured services was the protocol for the treatment of intracranial germinoma, a recently published Brazilian consortium that demonstrated with a median follow-up of 44.5 months, overall and event-free survivals of 100% (29, 30).

Another process to be filled in the task of diagnosis and treatment of pediatric brain tumors is to organize the referral pathways and the length during treatment. Diaz-Coronado et al, described that a child with medulloblastoma starting radiotherapy more than 30 days after surgery showed a negative impact on their survival (29).

An important element to provide comprehensive, context-related care is the existence of cooperative research groups. In Central America, the AHOPCA (Central American Association of Pediatric Hematology and Oncology) for the last 20 years which has unified countries and applied risk-adapted protocols and established patient registries. In addition, the Latin American Pediatric Oncology Group (GALOP) that have supported the conduct of local clinical trials in solid tumors (Ewing sarcoma, retinoblastoma, osteosarcoma). These are initiatives needed to narrow the treatment gap (7, 17).

Quantitative needs assessments for institutions in Brazil, Paraguay, and Chile were performed to provide an understanding of their strengths and their challenges. These play an important role to develop guidelines or policies that can help reduce the gaps in terms of resources (31–33). In the context of the molecular transformation to the field of pediatric neuro-oncology, access to an optimal pathology service is key to inform optimal treatment strategies (34). A comprehensive evolution of neuro-pathology services in Latin America, unfortunately does not exist. For children with CNS tumors, access to timely radiotherapy is essential to achieve optimal outcomes. In Latin America and the Caribbean in 2018, 30% (12/40) of countries did not have any radiotherapy centers, and only 7.5% (3/40) of countries (Antigua/Barbuda, Curacao and Uruguay) met the International Atomic Energy Agency’s (IAEA) recommendation of 1 megavoltage machine (MVM) per 250,000 inhabitants (35).

The expertise advisory programs, such as in surgical procedures, or web-based tumor boards, can dramatically change a patient’s survival. The LATB (Latin American Brain Tumor Board) reviews patients weekly with multidisciplinary teams from South and Central America, providing timely therapeutic recommendations, and continuous education, while also recognizing the institutional resource limitations across the different regions (14, 33, 36). Also, The Global Alliance for Pediatric Neuro-Oncology (GAP-NO), built from a pediatric neuro-oncology course focusing on LMIC needs, is another step forward in education of professionals, creating a network, and building capacity through research and national policies (37).

Developing and implementing policies that prioritize palliative care for pediatric brain tumors is an ongoing effort. Advocacy for policy changes at the national and regional levels is important to ensure sustainable and widespread access to palliative care services. It’s important to recognize the progress being made in Latin America while acknowledging the need for continued efforts to enhance palliative care services for children with brain tumors and other life-limiting conditions. Increased awareness, education, and collaboration are key factors in improving the overall quality of care for affected children and their families in the region (27, 38–40).

The importance of recognizing the educational gap in pediatric neuro-oncology among healthcare providers was described in a global cross-sectional survey demonstrating the importance of raising professional awareness on knowledge about clinical presentations of CNS tumors (41). Since 2020 an expert panel from Hospital Garrahan in Argentina aim to improved treatment in almost all centers in the country reaching to 96% of patients with brain tumors. This local intervention through monthly multidisciplinary virtual meetings between private and public hospitals has the aim to reduce mortality and improved survival (42).

Global educational initiatives like the Neuro-Oncology Training Seminars launched by St. Jude Global in 2018 are helping to cultivate an understanding of the importance of a multidisciplinary approach to the management of children with brain tumors; in 2022 the program enrolled from 20 countries (43). Another example is the Pediatric Neuro-Oncology Virtual Fellowship which started in 2022 by St. Jude Global and has partnered 5 pediatric oncologists with local and international experts from pediatric neuro-oncology centers of excellence who mentor them and provide individualized training with a set curriculum. This ambitious project tries to reduce the knowledge gaps by providing access to sub-specialty training in pediatric neuro-oncology not otherwise offered locally.

Increasing educational knowledge capacity to improved diagnosis of brain tumors can be challenge, needs of a well structure and sustainable curriculum to be adopted by the health care workers on each country, meanwhile WHO and PAHO, implemented a virtual and free material on their web page to improved early diagnosis. In Peru there is a mobile application, called ONCOPEDS, that aims to facilitate early diagnosis and education not only to health care providers, but parents and teachers. ONCOPEDS has promising results so far improving the referral time and education in Peru (44).

There are undoubtedly challenges ahead to narrow the gap between HIC and LMIC of pediatric oncology and specially neuro-oncology, but certainly, with joint efforts, the survival disparity will be narrowed. Initiatives in Latin America are already being implemented and paving the way to inform other regions of optimal strategies to build care capacity to improve outcomes for children with CNS tumors.

## Author contributions

RD-C: Conceptualization, Validation, Visualization, Writing – original draft, Writing – review and editing. RC-V: Validation, Visualization, Writing – original draft, Writing – review and editing. AC: Conceptualization, Supervision, Validation, Visualization, Writing – original draft, Writing – review and editing.
